# Seismic Behavior of Existing Reinforced Concrete Columns with Non-Seismic Details under Low Axial Loads

**DOI:** 10.3390/ma15031239

**Published:** 2022-02-07

**Authors:** Myeong-Ho Choi, Chang-Hwan Lee

**Affiliations:** 1 Division of Architectural and Fire Protection Engineering, Pukyong National University, 45, Yongso-ro, Nam-gu, Busan 48513, Korea; audgh39@pukyong.ac.kr; 2 Department of Architectural Engineering, Pukyong National University, 45, Yongso-ro, Nam-gu, Busan 48513, Korea

**Keywords:** reinforced concrete columns, non-seismic detail, transverse reinforcement, hook angle, low axial load, seismic performance, strain behavior, ductility, energy dissipation

## Abstract

Reinforced concrete (RC) columns of old existing buildings are vulnerable to earthquakes because the hoops comprising their transverse reinforcement are widely spaced and anchored using 90° hooks. This study extensively evaluated the seismic behavior of RC columns with such non-seismic details. Experiments were conducted by applying lateral cyclic loads to five full-scale column specimens with various transverse reinforcement details subjected to low axial loads. The experimental results demonstrated that the internal transverse crosstie had a significant confinement effect in the non-seismic detailed columns with 90° hoop anchor hooks. In addition, the lateral load–drift relationships, ductilities, and energy dissipation capabilities of the columns were not significantly affected by the hoop spacing or anchor hook angle when a low axial load was applied up to a drift ratio of 3.5% before failure. The evaluation model based on ASCE/SEI 41-17 was then shown to approximate the initial stiffness, maximum strength, and post-peak strength reduction behavior of the non-seismically reinforced column. This study was based on the experimental behavior of single column members, and it needs to be extended to research on frame structures in which columns are connected to beams and slabs.

## 1. Introduction

Reinforced concrete (RC) buildings constructed before the application of seismic design standards or in areas with low earthquake prevalence mostly have non-seismic details that are vulnerable to earthquake damage. Such details include the use of 90° hooks to close the transverse hoops or overall wide spacing of transverse reinforcement in columns. A similar observation has been made in small one- or two-story RC buildings in which the columns are subjected to low compressive forces [1]. These types of columns are considered to have lower lateral load resistance and inelastic deformation capacities compared with the levels required by the current seismic design standards.

The results of previous studies on RC buildings with non-seismic details have indicated that the weak reinforcement of columns is the main cause of a weak column–strong beam structure, which can lead to an entire building exhibiting a weak response to earthquakes [2,3]. In addition, though column shear failure is desirable after flexural yielding, shear failure can occur before flexural yielding in columns with long-term accumulation of damage and/or inadequate seismic details. Experimental studies using the standard code at the time of design have shown that the transverse reinforcement of columns with weak reinforcement did not meet the current standard code requirements to prevent shear failure [4,5].

To address these problems, the provisions for earthquake-resistant structures outlined in ACI 318-19 [6] require the transverse reinforcement of a column to be anchored with 135° hooks and set stricter provisions on spacing. Many studies have been conducted to upgrade the seismic performance of existing RC buildings [7,8,9]. Analytical models for RC elements have been developed to improve the accuracy of seismic evaluation [10], and theoretical and numerical approaches for damage modeling of concrete structureshave also been discussed [11,12,13]. Nevertheless, on-site implementation is difficult to achieve, and operations may not be properly conducted if the construction supervisor does not perform sufficient checks, resulting in issues such as poor quality of the 135° hooks in the transverse shear reinforcement and inconsistent spacing between transverse reinforcement even in recent years [14]. These construction conditions suggest that the columns of old RC buildings may be especially vulnerable to earthquakes.

Accordingly, various studies have been conducted to investigate the behavior of RC columns with non-seismic details. Studies on the effect of axial load have shown that the ductility of an RC column decreases as the axial load ratio applied to it increases [15,16]. Similar results were found in experiments on concrete columns using low-quality concrete and high-strength materials [17,18,19]. A study conducted by Mo and Wang showed that the spacing of transverse reinforcement had a significant effect on the performance of the column [20]. However, it was confirmed that the ductility of the RC column was more sensitively affected by the axial force than the cross-sectional details [21,22,23].

Lam et al. conducted experiments on 1/3-scale RC columns with non-seismic details using axial load, shear span-to-depth ratio, and transverse reinforcement hook angle as variables. The results confirmed that the deformation capacity decreased with decreasing shear span-to-depth ratio and increasing axial load ratio, and that the deformation capacity was 40% lower when the anchor hook angle was 90° than when it was 135° [24]. Kim et al. experimentally evaluated the influence of axial load, longitudinal reinforcement, and transverse reinforcement anchor hook angle on 1/2-scale RC columns with non-seismic details. The results also showed that the larger the applied axial load, the smaller the energy dissipation due to a rapid reduction in strength after the maximum load was achieved. However, because the transverse reinforcements were placed with a rather large spacing of 300 mm, no difference was observed in the strains in the transverse reinforcement (i.e., concrete confinement) in the parallel direction according to different anchor hook angles, though it was noted that additional research needs to be conducted to investigate this observation [25]. Another study by Kim et al. on RC columns subjected to low compressive forces reported that column specimens designed using hoops anchored with 90° hooks exhibited the same load-carrying capacity as column specimens designed using hoops with 135° anchor hooks, but failure occurred earlier in the column with 90° hooks [26]. This phenomenon can be explained by the loss of binding force at the 90° hook anchorage at the same time as the cover failure of the concrete, leading to a decrease in the transverse reinforcement and shear resistance capacities of the hoop. However, the experimental results showed that 90° hooks were sufficient for small buildings subjected to low axial forces and could be an appropriate detail in terms of constructability [26,27].

As mentioned above, the effect of hoop anchor hook angle on RC columns subjected to low compressive forces has been observed to vary depending on the axial force ratio and the spacing of transverse reinforcements, but further clarification of the effects corresponding to the changes in these variables is needed. Against this background, the seismic behavior of RC columns with non-seismic details subjected to low axial loads—such as typically found in old existing buildings—were thoroughly evaluated in this study. First, the details of cyclic experiments conducted on five full-scale column specimens are described. Then, the experimental results are analyzed and discussed in terms of the load–drift relationship, failure mode, strain behavior, ductility, and energy dissipation. Finally, a comparison with the evaluation model applied according to ASCE/SEI 41-17 is given.

## 2. Test Plan and Methods

### 2.1. Specimen Geometry and Configuration

**Figure 1:**
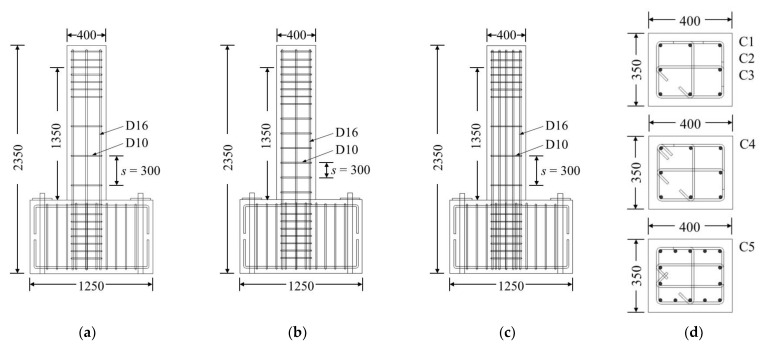
Details of longitudinal and transverse reinforcement for column specimens (all dimensions in mm): (**a**) C1, C2, and C4; (**b**) C3; (**c**) C5; (**d**) Cross-sections.

In this study, experiments were conducted on the exterior and interior columns of an RC building with non-seismic details. The column inflection point was assumed to be located in the middle of the clear height of the column segment (*h_n_*). Each column specimen was fabricated at full-scale to evaluate behavior identical to that of an actual column. The column cross-section had dimensions of 400 × 350 mm. The lateral load was applied 1350 mm above (i.e., *h_n_*/2) the top surface of the footing (Figure 1) in the strong axis direction.

The details of the specimens are summarized in Table 1, in which C2 is a reference specimen fabricated according to the general conditions at the time of construction, with a transverse reinforcement (hoop and crosstie) spacing *s* of 300 mm, 90° hoop anchor hooks, and a constant axial load of 0.1*A_g_f′_c_*; C1 had the same characteristics as C2 but no axial load was applied; C3 had the same characteristics as C2 but the value of *s* was reduced to 150 mm; the basic conditions of the cross-section in C4 were the same as in C1, C2, and C3, but with 135° anchor hooks on the hoops; C5 was designed by referring to the details of the existing interior column having the highest longitudinal reinforcement ratio (*ρ*) and axial load ratio. In all specimens, the internal crossties were designed to have a 90° hook on one end and a 135° hook on the other, as was commonly used in many existing buildings. The crosstie directions were alternated end for end so that the same hooks were not on adjacent levels.

### 2.2. Material Properties

To measure the material strength of the concrete used, a total of six 100 by 200 mm concrete cylinders were made in accordance with KS F 2403 [28]. They were cured under the same conditions as the specimens, and compressive tests on three cylinders were performed at the specimen’s first test date (28 days) and the final test date (32 days), respectively [29]. The compressive strength of concrete (*f′_c_*) was determined as the average of the values measured at the two dates; the *f′_c_* value was 16.12 MPa. Tensile tests for the rebar were performed in accordance with KS D 0802 [30]; the results indicated that the yield stresses (*f_y_*) in the D16 longitudinal bars and D10 transverse bars were 468.3 and 467.3 MPa, respectively.

### 2.3. Test Setup

**Figure 2:**
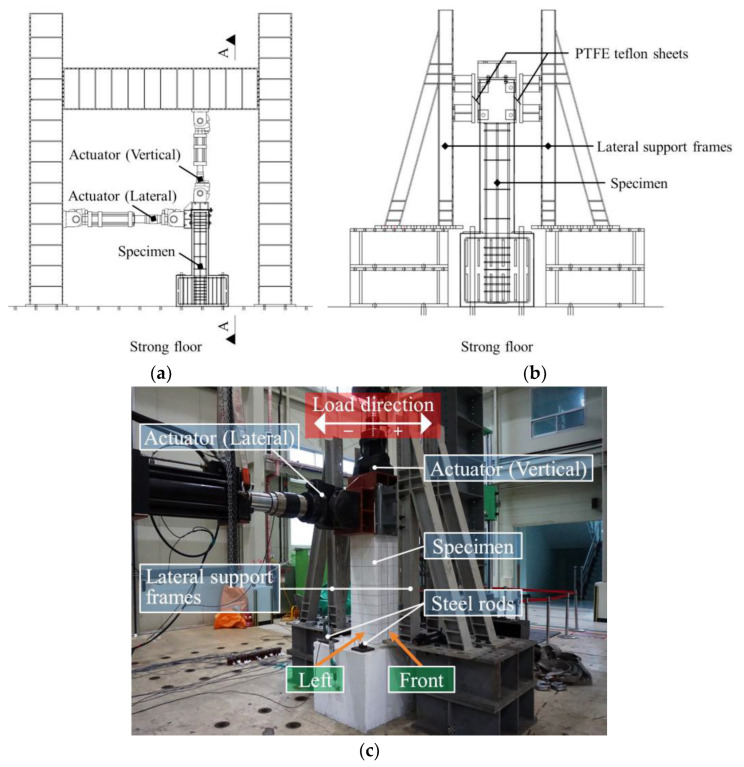
Test setup for applying vertical and horizontal loads to RC columns: (**a**) Front view; (**b**) Side view (section A-A); (**c**) Photograph.

Figure 2 shows the details of the test setup. To apply vertical and horizontal loads, actuators in two directions were connected to the top of the column specimens (Figure 2a). The loading tests were conducted by first applying the axial load in the vertical direction (excluding C1), followed by applying the cyclic loading in the horizontal (lateral) direction using a 1000 kN actuator (MTS Corporation).

The specimens were laterally supported to prevent out-of-plane behavior (Figure 2b), and frictional resistance between the specimen and the lateral support frames was minimized by installing PTFE teflon sheets. The footing was fixed on the strong floor using four steel rods, and the axial load acting on the column was maintained at a constant level using force control during cyclic lateral loading.

**Figure 3:**
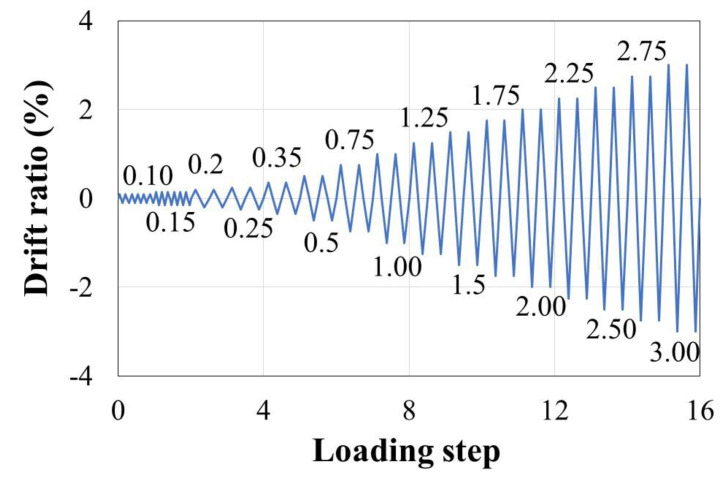
Drift history for cyclic loading test.

The cyclic lateral load applied to the specimens through displacement control was gradually increased, as shown in Figure 3 [31,32], and the drift ratio was calculated based on a column height of 1350 mm. Steps 1 and 2 in the initial loading process were each repeated for six cycles [33], and the steps beyond Step 3 were each repeated twice. After Step 16, loading was performed by increasing the drift ratio by 0.5%. The lateral load was continuously applied until it dropped below 70% of the maximum strength (*V_max_*) of the specimen.

### 2.4. Instrumentation

The axial and lateral loads acting on the columns were measured by load cells connected to the respective actuators. A separate linear variable differential transducer (LVDT) was installed at the height where the lateral load was applied to measure the lateral displacement of the specimen. Additional LVDTs were installed to determine whether horizontal sliding or vertical uplift occurred in the footing. Furthermore, several strain gauges were attached to the rebars in the plastic hinge zone of each column to measure the strains in the longitudinal and transverse bars during load application.

## 3. Results and Discussion

### 3.1. Load–Drift Relationships and Failure Modes

**Figure 4:**
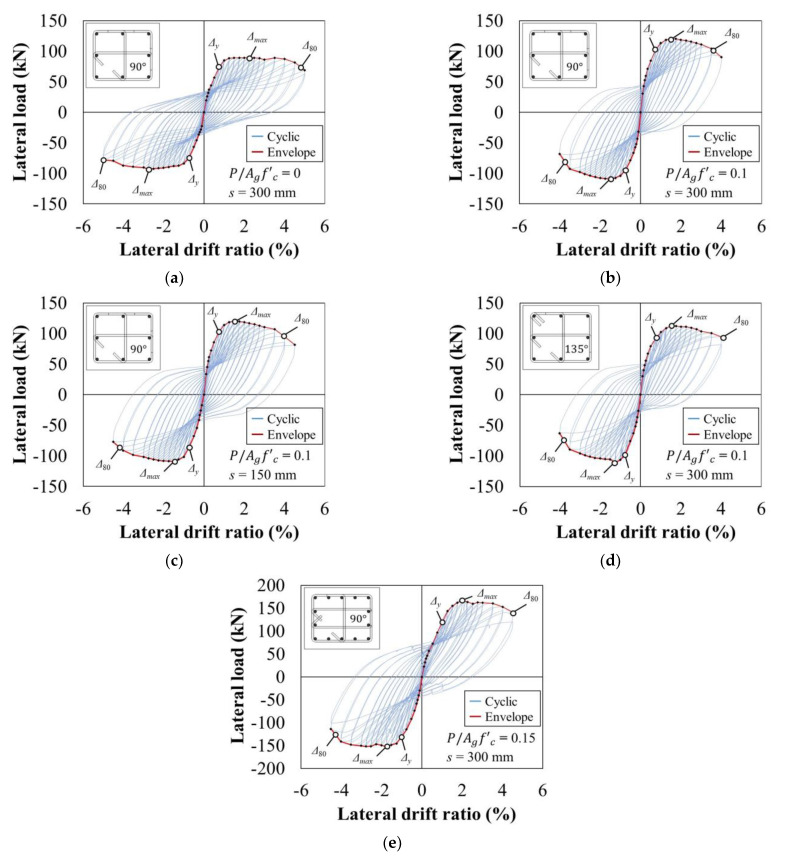
Lateral load–drift hysteresis curves of specimens: (**a**) C1; (**b**) C2; (**c**) C3; (**d**) C4; (**e**) C5.

Figure 4 displays the lateral load–drift hysteresis curves of all specimens, and the summarized results are listed in Table 2. For cyclic seismic testing of structural elements, relevant standards and guidelines specify the acceptance criteria. The point at which the acceptance criteria should be satisfied (i.e., the point of failure) is usually defined by the strength reduction ratio in terms of the maximum strength. In this study, the point of failure was determined by referring to ACI 374.2R-13 [34], the most relevant guide for the performed experiment. Specifically, the failure was defined to be occurring when the lateral load resistance was reduced by more than 20% after the specimen reached *V_max_* [34], and the pre-failure period was considered as the effective hysteresis [35].

**Figure 5:**
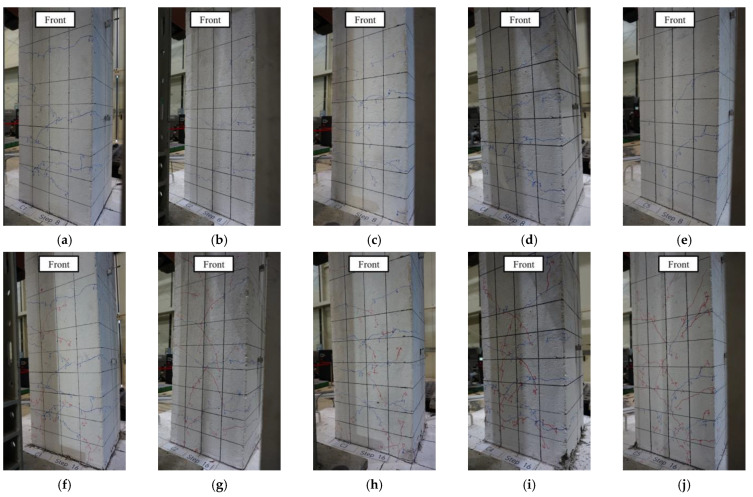
Crack pattern and damage state observed in the column before failure: (**a**–**e**) Step 8 (in the order of C1, C2, C3, C4, and C5); (**f**–**j**) Step 16 (in the order of C1, C2, C3, C4, and C5).

**Figure 6:**
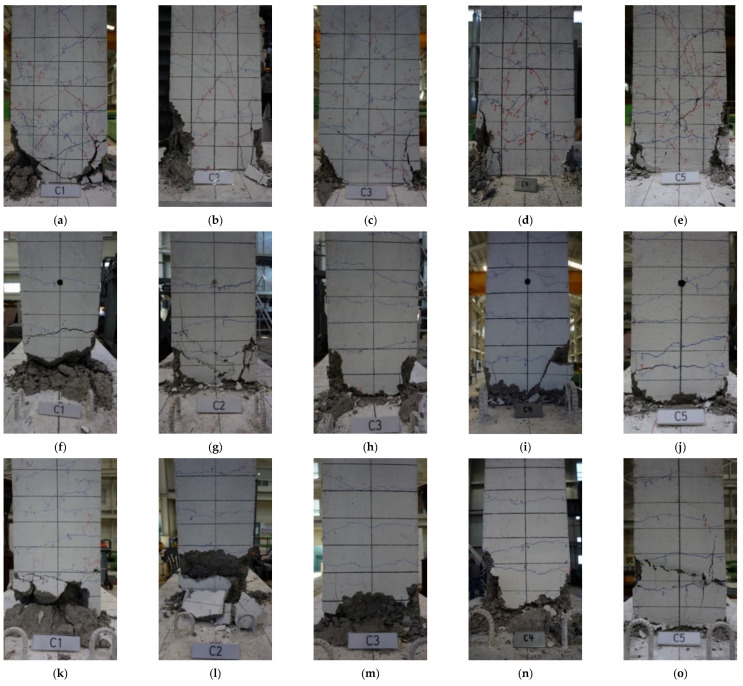
Final damage state observed on each side of the column at the end of the experiment: (**a**) C1, front; (**b**) C2, front; (**c**) C3, front; (**d**) C4, front; (**e**) C5, front; (**f**) C1, right; (**g**) C2, right; (**h**) C3, right; (**i**) C4, right; (**j**) C5, right; (**k**) C1, left; (**l**) C2, left; (**m**) C3, left; (**n**) C4, left; (**o**) C5, left.

The crack pattern and damage state of the columns in Step 8 (approximately when the longitudinal bars yielded) and Step 16 (immediately before failure) are shown in Figure 5. As can be seen from the figure, crack patterns did not differ significantly among specimens during the pre-failure load application. Figure 6 shows the damage state of each specimen at the end of the experiment (see Figure 2c for the faces of the columns). The results indicate that C1–C4 displayed a typical flexural failure mode accompanied by concrete crushing and cover spalling, whereas C5 exhibited shear failure after the longitudinal bars yielded.

Figure 4a shows the hysteresis of C1 with no axial loads applied. A transverse crack occurred on the side surface of the column when the lateral drift ratio was 0.2%, and *V_max_* values of +89.4 kN and −92.5 kN were recorded at lateral drift ratios of +2.25 and −2.75%, respectively. After reaching *V_max_* during cyclic loading, ductile behavior was observed without a significant decrease in strength and stiffness. At a lateral drift ratio of 4.0%, the strength sharply decreased with the crushing of the concrete cover at the end, and the experiment was finally terminated at a lateral drift ratio of 5.0% (Figure 6a,f,k).

In reference specimen C2 (Figure 4b), a transverse crack occurred on the side surface of the column when the lateral drift ratio was 0.35%, *V_max_* values of +121.0 and −109.3 kN were observed at a lateral drift ratio of ±1.5%, respectively, and shear cracks occurred in the plastic hinge zone. Cracks began to expand after reaching *V_max_* and widened at the joint connecting the column to the footing (i.e., the cold joint), resulting in a decrease in strength and stiffness. At a lateral drift ratio of 3.0%, the concrete cover was crushed with the expansion of vertical cracks on the side surface (Figure 6g), and the strength drastically decreased. Failure occurred at a lateral drift ratio of 3.5%, and the experiment was terminated at a lateral drift ratio of 4.0% (Figure 6b,g,l).

Figure 4c indicates that C3, which had a different transverse reinforcement spacing than the other specimens, showed nearly identical crack patterns and hysteresis to C2. Shear cracks occurred in the plastic hinge zone of the column at a lateral drift ratio of 0.75–1.0%, and *V_max_* values of +120.9 and −109.0 kN were recorded at ±1.5%, respectively. Similar behavior to C2 subsequently continued as the strength drastically decreased with the crushing of the concrete cover at a lateral drift ratio of 3.5%. Failure occurred at a lateral drift ratio of 4.0%, and the experiment was terminated at a lateral drift ratio of 4.5% (Figure 6c,h,m).

The hysteresis of C4 (Figure 4d), which used a 135° hook angle to anchor the hoops instead of the 90° angle used in the other specimens, also showed similar behavior to the reference specimen C2: *V_max_* values of +113.0 and −111.2 kN were observed at lateral drift ratios of +1.5 and −1.75%, respectively, and shear cracks expanded in the plastic hinge zone. The strength rapidly decreased with the crushing of the concrete at a lateral drift ratio of 3.0%. Failure occurred at the first 4.0% lateral drift ratio cycle, and the experiment was terminated in the second 4.0% cycle (Figure 6d,i,n).

Figure 4e shows the hysteresis of C5, which was designed as an interior column with a high axial load ratio and *ρ*. A transverse crack occurred on the side surface when the lateral drift ratio was 0.25%, shear cracks appeared in the plastic hinge zone at a lateral drift ratio of 1.25%, and *V_max_* values of +167.1 and −152.6 kN were observed at lateral drift ratios of +2.0 and −1.75%, respectively. The shear cracks subsequently expanded and the strength showed a decreasing tendency before the column exhibited ductile behavior once more. The strength drastically decreased at a lateral drift ratio of 3.5%, and the width of the shear cracks increased until failure occurred at a lateral drift ratio of 4.5%, terminating the experiment (Figure 6e,j,o).

### 3.2. Strain Behavior

**Figure 7:**
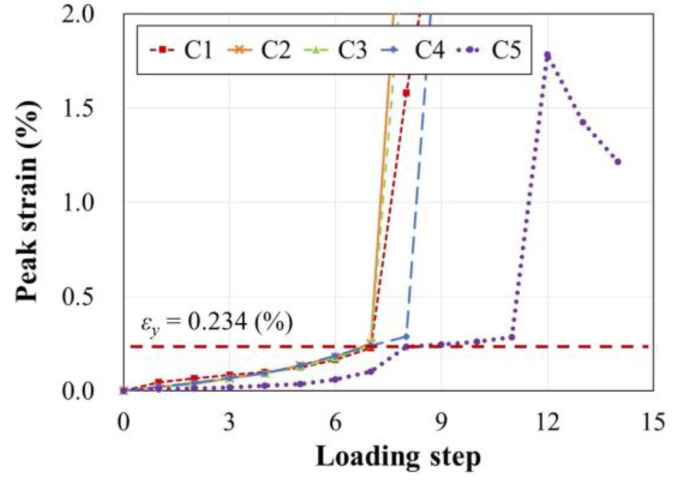
Peak strains in longitudinal bars.

Figure 7 shows the peak strains in the longitudinal bars of each specimen at each loading step. The yield strain (*ε_y_*) of the D16 bars used as the longitudinal bars was 0.00234. For C1, C2, C3, and C4, flexural cracks occurred on all four sides of the column before the longitudinal bar yielded (refer to Figure 5a–e). The bar yielded in Step 7 when shear cracks occurred on the front surface of the columns. For C5, which had a higher axial load ratio and *ρ*, the yielding occurred in Step 8. The maximum strain was recorded in Step 12 when *V_max_* was reached, beyond which the peak strains showed a decreasing tendency with gradually reducing strength of the column.

**Figure 8:**
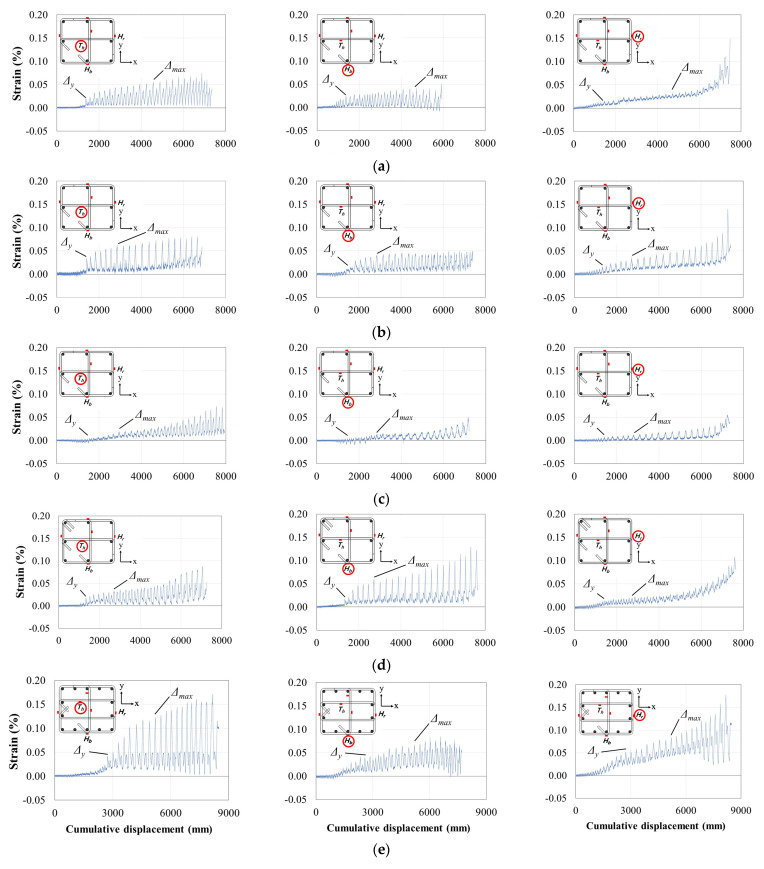
Strain distributions of transverse reinforcement (in the order of *T_h_*, *H_b_*, and *H_r_*): (**a**) C1; (**b**) C2; (**c**) C3; (**d**) C4; (**e**) C5.

Figure 8 shows the strain distributions in the transverse reinforcements located at a distance of *s*/2 from the bottom of the column, and the strain values recorded at Δ*_y_* and Δ*_max_* are summarized in Table 3. In the figure, *T_h_* denotes the strain measured at the internal crosstie (parallel to the lateral load), and *H_b_* and *H_r_* denote the strains measured at the bottom of the hoop (parallel to the lateral load) and the right side of the hoop (perpendicular to the lateral load), respectively. All specimens excluding C3, which had an *s* value of 150 mm, showed a significant increase in *T_h_* with increasing confinement effect provided by the transverse reinforcement once the longitudinal bars yielded. However, the strains measured in the hoop (i.e., *H_b_* and *H_r_*) steadily increased regardless of the yielding point of the longitudinal bars.

Meanwhile, a difference in the strain behaviors of *H_b_* and *T_h_* was observed in the direction of the lateral load. The specimens using 90° hoop anchor hooks (C1, C2, C3, and C5) showed larger strains in the internal crosstie than in the hoop. This is presumed to happen because one side of the crosstie was anchored using a 135° hook, which induced a higher confinement effect than the hoops anchored using 90° hooks. The confinement effect of the hoop was further increased in C4, which had closed hoops anchored using 135° hooks. From these results, the internal crosstie was confirmed to exert a significant confinement effect in non-seismic detailed columns using 90° hooks to anchor the hoops.

The values of both *H_b_* and *T_h_* were affected by shear forces and confinement effects, while the measured *H_r_* values were not affected by shear forces, implying that the value of *H_r_* may be relatively small. The hoops anchored using 135° hooks in C4 provided excellent shear force resistance and confinement, resulting in the expected strain distribution. However, the values of *H_r_* (perpendicular to the lateral load) in C1, C2, and C5 were similar to or larger than those of *H_b_* (parallel to the lateral load). Furthermore, C2 exhibited a wider range of vertical cracks and concrete spalling expansion in the front and side surfaces of the column than C4 (see Figure 6), suggesting larger deformation of the longitudinal bars and hoops owing to the lower confinement effect of the hoops anchored using 90° hooks.

### 3.3. Ductility

**Figure 9:**
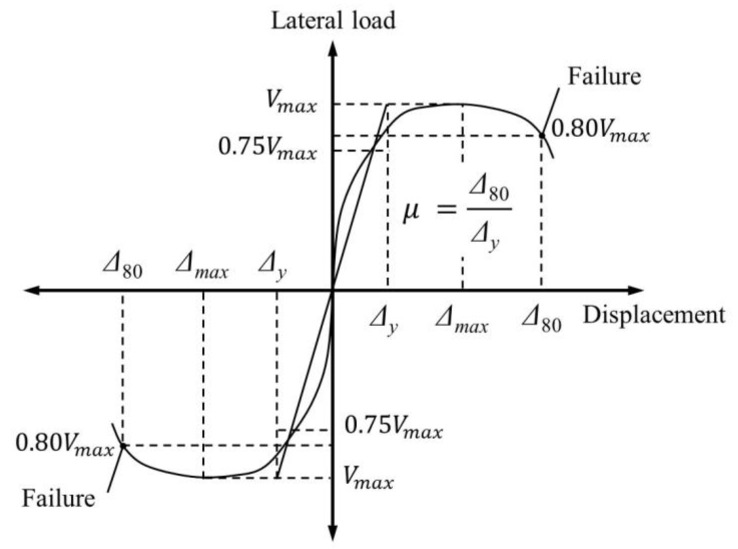
Definition of structural characteristics (structural yielding, failure, and ductility).

Ductility factor was calculated using Equation (1) to compare the ductilities of the column specimens with varying details in this study [36].

(1) $$\mu =\frac{\Delta_{80}}{\Delta_{y}}$$

where Δ_80_ is the displacement at the point of failure when the load is reduced to 0.8*V_max_*, and Δ*_y_* is the calculated displacement at the point where the secant line at a load of 0.75*V_max_* intersects with the horizontal line at the y-axis value of *V_max_* on the load–displacement curve. Figure 9 illustrates the definitions of these structural characteristics on the cyclic envelope curve, and the corresponding values are summarized in Table 2. The value of *V_y_* that corresponds to Δ*_y_* was obtained through experiments in this study (Table 2).

**Figure 10:**
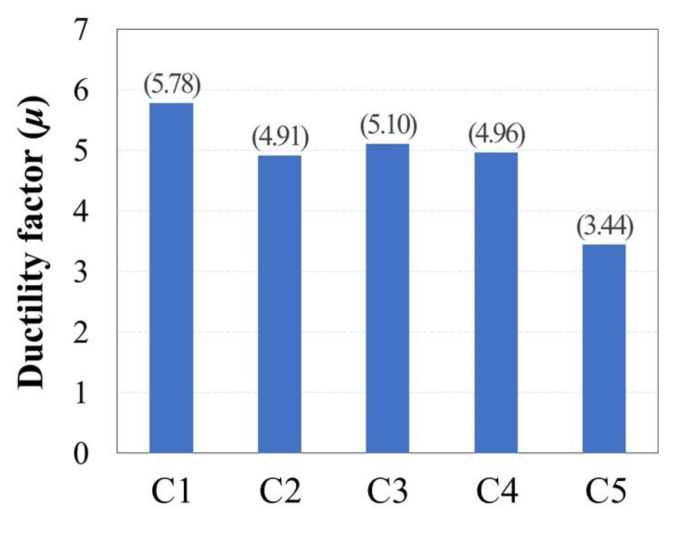
Comparison of ductility factors (*μ*).

Figure 10 shows a bar graph comparing the values of *μ* for the different evaluated specimens; all specimens show a *μ* value greater than 3, generally exhibiting ductile behavior. Since no axial load was applied to C1, its *μ* was 5.78, or 17.7% higher than that of reference specimen C2. Indeed, C1 exhibited the most ductile behavior of all the specimens. The *μ* value of C3, which had an *s* value of 150 mm, was 3.9% higher than that of C2 owing to the strong influence of the volumetric ratio of transverse reinforcement. Specimen C4 employed hoops anchored with 135° hooks but showed similar ductility to C2, which employed hoops anchored with 90° hooks. These results confirm that the volumetric ratio of transverse reinforcement exerts a greater effect on *μ* than the angle of the hoop anchor hooks in buildings subjected to low axial load. Finally, specimen C5 was designed to provide a *ρ* value 76.1% higher than other specimens and showed a *μ* value 42.7% lower than C2, indicating the least ductile behavior.

### 3.4. Energy Dissipation

**Figure 11:**
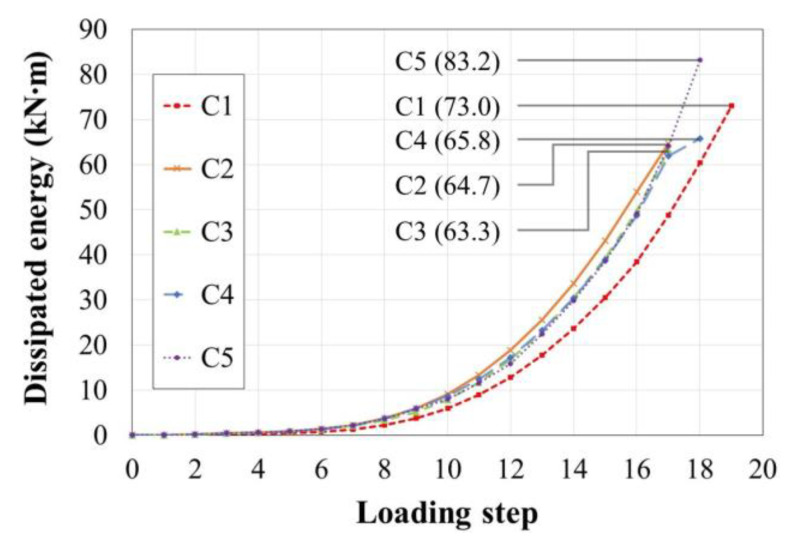
Cumulative dissipated energy until failure.

In addition to ductility, energy dissipation capacity is considered an important factor when evaluating the seismic performance of structural components. The total dissipated energy (*E_t_*) was therefore calculated by summing the area enclosed by the valid load–displacement hysteresis before failure, with the results listed in Table 2 and shown in Figure 11.

More energy was dissipated at the same loading step in C2 (to which an axial load of 0.1*A_g_f′_c_* was applied) than in C1 (to which no axial load was applied) owing to the associated increase in shear strength. However, C1 exhibited greater deformation capacity, resulting in an *E_t_* value 12.8% higher than that of C2. Though C3 and C4 initially dissipated less energy than C2, the failure points were the same or similar to C2, resulting in no significant difference in *E_t_* among the three specimens. The ascending slopes of C3 and C4 were confirmed to be significantly larger than that of C2 at Step 17. This occurred because while the confinement effect drastically decreased from the initiation of cover failure in C2, the confinement effect of the transverse reinforcement was greater in C3 (which had half the *s* of 150 mm) and C4 (which had 135° hoop anchor hooks) even when the cover partially failed, implying that C3 and C4 provide stable energy dissipation. Note that if the experiment continued beyond the failure point specified in this study, the amount of energy dissipated in C3 and C4 would have drastically exceeded the amount in C2 at the same step.

Based on the above analysis, the smaller value of *s* and 135° hoop anchor hook detail were confirmed to enhance the ductile behavior at ultimate levels more than those of non-seismic RC columns, as is well known. However, this study focused on the seismic behavior of existing non-seismic RC columns subjected to low axial loads, and all specimens stably satisfied the minimum drift ratio of 3.5% before failure, considerably exceeding the allowable story drift ratio for seismic evaluations of existing RC buildings and indicating that existing columns with non-seismic details can exhibit sufficient seismic performance without individual reinforcement when subjected to low axial load conditions.

### 3.5. Comparison of Experimental Curves with the Evaluation Model Provided in ASCE 41

In order to evaluate an existing building to which seismic design is not applied, it must be checked whether structural elements have the required seismic performance based on the structural analysis results. ASCE/SEI 41-17 [37] provides a standard for seismic evaluation and retrofit of existing buildings, which is widely used in related research [38,39,40]. In this section, the experimental results are compared with the evaluation model for concrete columns of ASCE 41-17.

**Figure 12:**
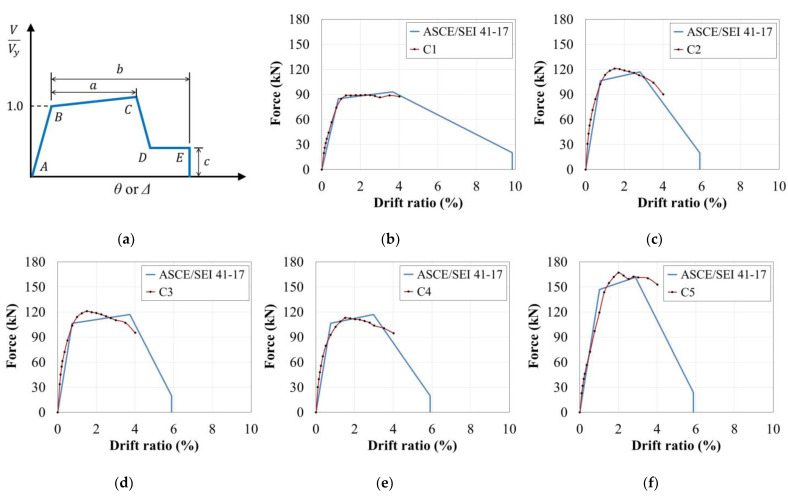
Comparison of backbone curves obtained from ASCE/SEI 41-17 and the test results: (**a**) Generalized component force–deformation relations; (**b**) C1; (**c**) C2; (**d**) C3; (**e**) C4; (**f**) C5.

A generalized evaluation model of structural elements is proposed in ASCE/SEI 41-17, as shown in Figure 12a. The section *AB* corresponding to linear behavior is determined by the type and condition of the components, among which the effective rigidity of RC columns is calculated according to Table 4.

The distances *a* and *b* in Figure 12a are parameters required to define the non-linear region and are used to calculate the plastic rotation angle, to points *C* (where strength begins to decrease beyond *V_y_*) and *E* (corresponding to collapse), respectively. For columns not controlled by inadequate development or splicing, the parameter *a* is calculated by Equation (2).

(2) $$a=\left(0.042-0.043\frac{N_{u}}{A_{g}f\prime_{c}}+0.63\rho_{t}-0.023\frac{V_{y}}{V_{o}}\right)$$

where *N_u_* is the axial load applied on the column section, and *ρ_t_* is the ratio of area of distributed transverse reinforcement to gross concrete area perpendicular to that reinforcement. *V_o_* is the nominal shear strength of concrete columns, which can be calculated using Equation (3).

(3) $$V_{o}=k_{nl}\left[\frac{A_{v}f_{y}d}{s}+\lambda \left(\frac{0.5\sqrt{f\prime_{c}}}{M/Vd}\sqrt{1+\frac{N_{u}}{0.5\sqrt{f\prime_{c}}A_{g}}}\right)0.8A_{g}\right]$$

where *k_nl_* is the coefficient used to calculate column shear strength based on displacement ductility. This value is 1.0 when displacement ductility demand is less than or equal to 2 and is 0.7 when ductility is greater than or equal to 6. In this study, the value of *μ* calculated in Section 3.3 was used as the displacement ductility. *M/Vd* is the largest ratio of moment to shear times effective depth (*d*) under design loadings (shall not be taken as greater than 4 or less than 2). The parameter *b* is calculated by Equation (4).

(4) $$b=\frac{0.5}{5+\frac{N_{u}}{0.8A_{g}f\prime_{c}}\frac{1}{\rho_{t}}\frac{f\prime_{c}}{f_{y}}}-0.01\geq a$$

The performances of column components tend to be incorrectly evaluated when using a model that shows a sharp decrease in strength from point *C* to *D* in Figure 12a unless a sudden decrease in strength appears during the corresponding experiment. For this reason, a linear decrease from point *C* to *E* is depicted to represent a more realistic behavior.

The backbone curves of the column specimens obtained by the above method were compared with the experimental results as shown in Figure 12b–f. Comparing these results demonstrates that the initial stiffness obtained using the standard model was similar to that obtained from the experimental results for all specimens. Note that it was impossible to compare the strength and deformation at extreme levels because the loading in this experiment only proceeded up to the point when the strength was reduced to less than 70% of *V_max_*. However, when considering only the tested range, the peak strength predicted by the standard model and the corresponding strength reduction were generally similar to the experimental results. Based on this finding, the modeling approach presented in ASCE/SEI 41-17 accurately represents the inelastic behavior of column components when preceded by flexural yielding, as in the specimens evaluated in this study. Nevertheless, C5, a column specimen with high axial load and *ρ*, was evaluated more conservatively by ASCE/ SEI 41-17.

## 4. Conclusions

Existing RC buildings to which seismic design is not applied have non-seismic details that are vulnerable to earthquake damage. In particular, the brittle behavior of the columns may cause the collapse of the entire building during an earthquake, so careful attention is required. In this study, five full-scale specimens (C1–C5) were subjected to a constant axial load and increasing cyclic lateral load to evaluate the seismic behavior of RC columns with non-seismic details designed to resist only small compressive forces. The following conclusions were obtained.

(1) After the longitudinal bars yielded, a shear failure occurred in C5, which was designed and tested as a high axial load column. However, the other specimens (C1–C4) showed typical flexural failure modes accompanied by concrete crushing and cover failure, indicating that RC columns with non-seismic details subjected to low axial load can exhibit ductile behavior.
(2) The strain in the internal crosstie, which had a 90° hook on one end and a 135° hook on the other, was larger when the hoop anchor hook angle was 90° than when it was 135°. In contrast, the confinement effect of the hoops increased when the hoop anchor hook angle was 135° (C4), leading to a decrease in strain in the crosstie. Therefore, the internal crosstie was confirmed to exert more influence on the confinement effect in non-seismic detailed columns with 90° hoop anchor hooks.
(3) Specimens C3 (with half the spacing of 150 mm between transverse reinforcements compared with the other specimens) and C4 (with 135° hoop anchor hooks) both showed similar load–drift curves as the reference specimen, C2, but slightly higher ductility ratios. Specimens C3 and C4 were expected to exhibit more ductile behavior at ultimate load levels beyond failure, but the energy dissipated in the three specimens up to failure was relatively similar. Therefore, existing columns with non-seismic details are expected to display considerable seismic performance without specific reinforcement measures when only a low axial load is applied.
(4) The evaluation model based on ASCE/SEI 41-17 predicted values for the initial stiffness, maximum strength, and strength reduction trend beyond the peak load that were similar to the experimental results. The model was thus deemed to accurately represent the inelastic behavior of RC column components when preceded by flexural yielding, such as in the specimens evaluated in this study.

Based only on the experimental behavior of single column members, the above conclusions were drawn in this study. However, other structural elements such as beams, slabs, and walls interact with the columns, affecting the seismic behavior of the columns. Therefore, further studies on existing RC columns with non-seismic details are recommended concerning frame and wall-frame structures.

## Figures and Tables

**Table 1:** Details of column specimens.

Specimen	Longitudinal Reinforcement		Transverse Reinforcement	Hook Angle (Hoop)	Applied Axial Load
C1	8-D16	(*ρ* = 0.0113)	D10@300	90°	0
C2	8-D16	(*ρ* = 0.0113)	D10@300	90°	0.1*A_g_f′_c_*
C3	8-D16	(*ρ* = 0.0113)	D10@150	90°	0.1*A_g_f′_c_*
C4	8-D16	(*ρ* = 0.0113)	D10@300	135°	0.1*A_g_f′_c_*
C5	16-D16	(*ρ* = 0.0199)	D10@300	90°	0.15*A_g_f′_c_*

Note: *A_g_* is the gross area of concrete section.

**Table 2:** Summary of test results.

Specimen	*V_y_*		*V_max_*		Δ*_y_*		Δ*_max_*		*μ*	Dissipated Energy (kN∙m)
	Pos	Neg	Pos	Neg	Pos	Neg	Pos	Neg		
C1	67.0	−69.4	89.4	−92.5	8.61	−9.04	30.4	−37.2	5.78	73.0
C2	90.7	−81.9	121.0	−109.3	7.79	−7.64	20.3	−20.3	4.91	64.7
C3	90.7	−81.7	120.9	−109.0	7.60	−9.04	20.4	−20.4	5.10	63.3
C4	84.7	−83.4	113.0	−111.2	7.90	−7.31	20.5	−17.0	4.96	65.8
C5	125.3	−114.5	167.1	−152.6	13.73	−10.69	13.7	−10.7	3.44	83.2

Note: *V_y_* is the yield strength, Δ*_y_* is the yield displacement, Δ*_max_* is the displacement where *V_max_* was reached, and *μ* is the ductility factor.

**Table 3:** Summary of recorded strain values at Δ*_y_* and Δ*_max_*.

Specimen	*T_h_*		*H_b_*		*H_r_*	
	Δ*_y_*	Δ*_max_*	Δ*_y_*	Δ*_max_*	Δ*_y_*	Δ*_max_*
C1	0.019	0.056	0.026	0.041	0.015	0.032
C2	0.033	0.062	0.016	0.041	0.016	0.038
C3	0.003	0.019	0.004	0.013	0.007	0.012
C4	0.018	0.033	0.017	0.060	0.014	0.020
C5	0.042	0.123	0.040	0.067	0.052	0.084

**Table 4:** Effective rigidity values (ASCE/SEI 41-17 [37]).

Component	Flexural	Shear	Axial
Columns with compression caused by design gravity loads ≥ 0.5*A_g_f′_c_*	0.7*E_c_I_g_*	0.4*E_c_A_w_*	*E_c_A_g_*
Columns with compression caused by design gravity loads ≤ 0.1*A_g_f′_c_* or with tension	0.3*E_c_I_g_*	0.4*E_c_A_w_*	*E_c_A_g_* (compression) *E_s_A_s_* (tension)

Note: *E_c_* is the modulus of elasticity of concrete column, *E_s_* is the modulus of elasticity of reinforcement, *I_g_* is the moment of inertia of gross concrete, *A_w_* is the summation of the net horizontal cross-sectional area for concrete, and *A_s_* is the area of nonprestressed tension reinforcement.

## Data Availability

The data presented in this study are available upon request from the corresponding author. The data are not publicly available due to privacy.
